# Supplementary material for: Pastoralism may have delayed the end of the green Sahara
Source: Nat Commun. 2018 Oct 1;9:4018. doi: 10.1038/s41467-018-06321-y (PMC6167352; doi:10.1038/s41467-018-06321-y)
Supplement: Supplementary file 1 — Supplementary Information [file 41467_2018_6321_MOESM1_ESM.pdf]

Supplementary Table 1. Sampling ranges for model parameters

Parameter	Minimum Value	Maximum Value	Value used in Supplementary Figure 1
a	-3	3	0
b	-150	0	-100
c	0	5	2
d	0	2	1
τ_v	1	10	5
τ_N	1	10	5
σ	0	2	1

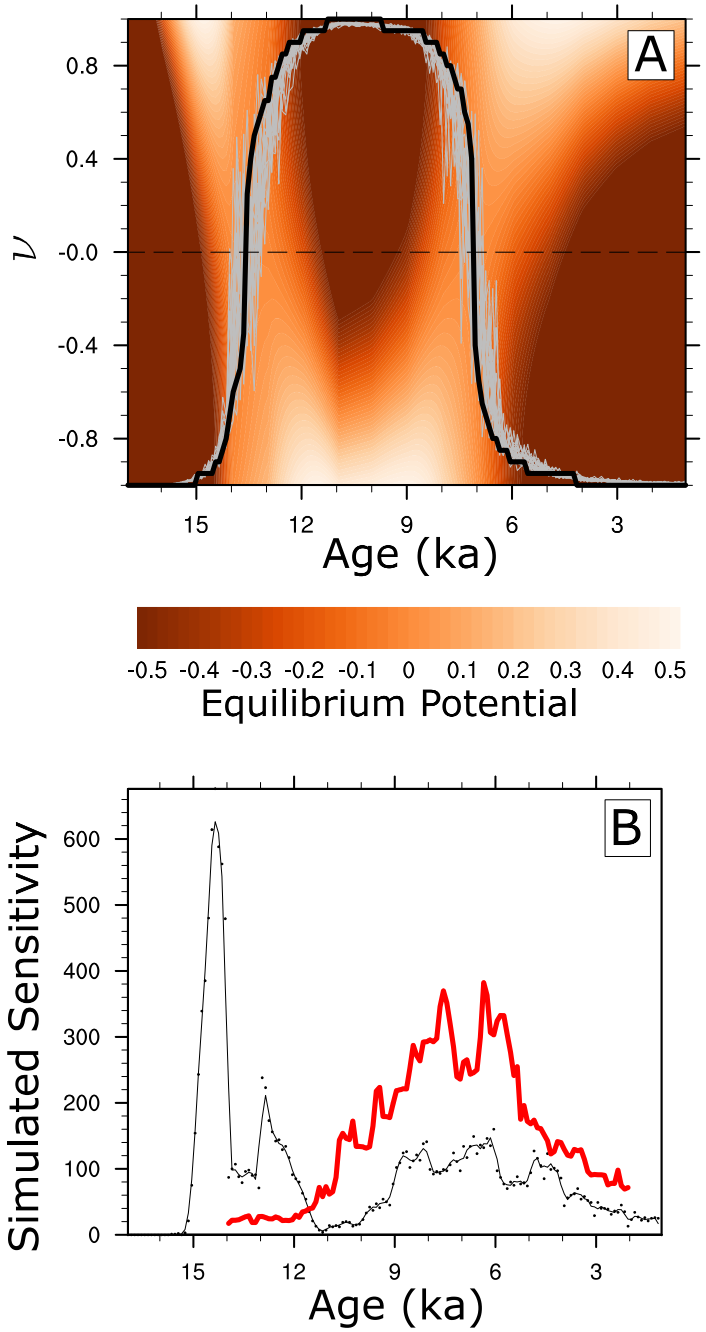

Supplementary Figure 1. The simulated sensitivity metric. (A) The equilibrium potential, $U(\nu)$, of a particular model instance (see Supplementary Table 1 for parameter settings). The minimum potential in each century (black) is shown along with the results of twenty fully stochastic simulations (gray). The threshold time calculated from eq. 7 occurs when the minimum potential (solid black) crosses the $\nu = 0$ line (dashed) (B) The simulated sensitivity of northern Africa diagnosed using the approach outlined in the Methods. Black dots show the number of not-implausible ensemble members with a threshold time in each century (black line is a 3 point running average). Also shown is the inferred population summed probability distribution (red; Manning & Timpson, 2014).

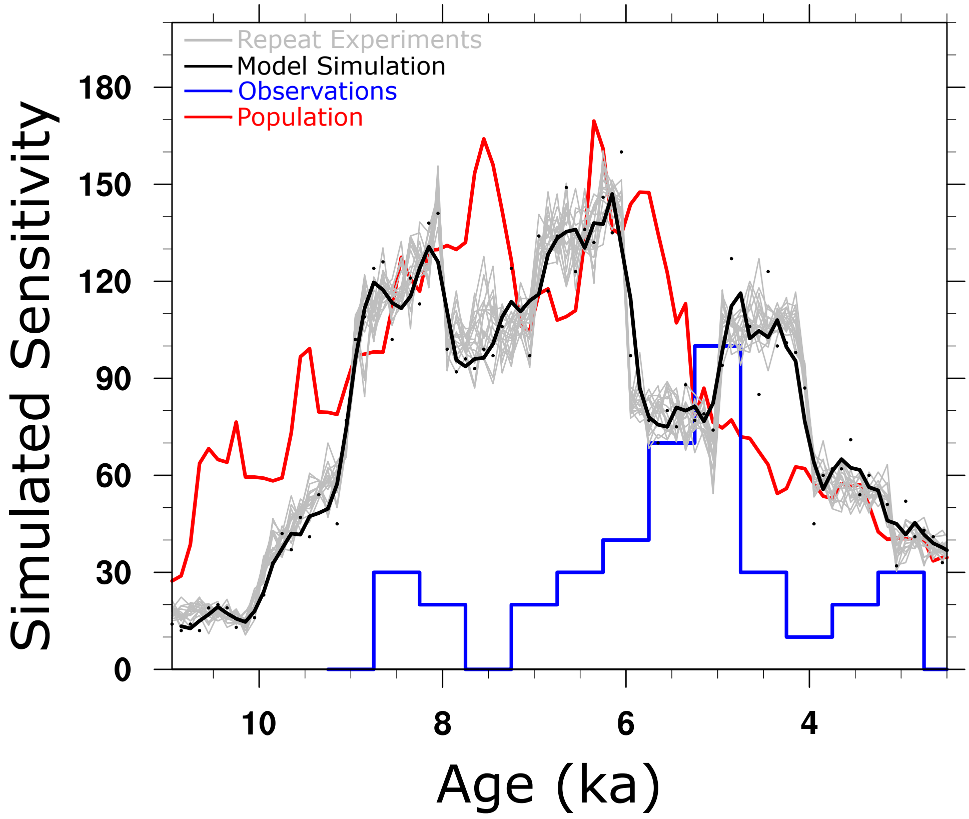

Supplementary Figure 2. The robustness of the model results. The simulated sensitivity (black), observed frequency of AHP end (blue, Shanahan et al., 2015) and reconstructed relative population (red; Manning & Timpson, 2014) are those shown in Fig. 5 (see fig 5 for axes scaling of the observations and population). The whole model experiment was replicated twenty times with different random sampling of the parameter ranges in Supplementary Table 1. The simulated sensitivities resulting from each of these replicates are shown in gray. They each show a similar temporal pattern with the dominant peak occurring just before 6000 years ago.
